# Get to know WONCA Africa

**DOI:** 10.4102/phcfm.v10i1.1984

**Published:** 2018-11-28

**Authors:** Shabir Moosa

**Affiliations:** 1 WONCA Africa, Johannesburg, South Africa; 2 Gauteng Department of Health, Johannesburg, South Africa; 3 Department of Family Medicine, University of the Witwatersrand, South Africa

As the new president of the World Organization of National Colleges, Academies and Academic Associations of General Practitioners/Family Physicians (WONCA) Africa, I realise that many family doctors in Africa know very little about WONCA. I hope that this editorial and other communications will begin to ensure every family doctor in Africa knows and supports WONCA. WONCA was set up in 1972. It built up a great brand value over the years. This was retained in developing a short name for the organisation: WONCA, the World Organisation of Family Doctors. WONCA Council is made up of 118 member organisations from colleges, academies and associations of family doctors in 131 countries across the world. The mission of WONCA is to improve the quality of life of people of the world through defining and promoting its values, including respect for universal human rights, including gender equity, and by fostering high standards of care in general practice/family medicine. It represents and acts as an advocate for its members at an international level where it interacts with world bodies such as the World Health Organization (WHO). A small WONCA Executive team meets regularly between biannual WONCA Council meetings. The 13 members of WONCA Executive team are the world president, president-elect, immediate past president, regional presidents from each of the seven WONCA regions – Africa, Asia Pacific, East Mediterranean, Europe, Iberoamericana-CIMF, North America and South Asia – and three members-at-large. The WONCA World Secretariat is currently located in Bangkok. WONCA has rich resources on its website, www.wonca.net. Subscribe to the newsletters and see the WONCA Africa page there at https://www.wonca.net/AboutWonca/Regions/Africa.aspx.

WONCA Africa Region is made up of ten paid-up member organisations as of October 2018: Association of Family Physicians of Uganda, Association of General and Private Medical Practitioners of Nigeria, College of Primary Care Physicians of Zimbabwe, Faculty of Family Medicine in the National Postgraduate Medical College of Nigeria, Kenya Association of Family Physicians, Lesotho Medical Association, Society of Family Physicians of Nigeria, South African Academy of Family Physicians, Society of Family Physicians of Ghana and the Faculty of Family Medicine (Ghana Chapter) in the West African College of Physicians. Africa was right there in WONCA in 1972. Recent presidents included Bruce Sparks, Khaya Mfenyana, Sylvester Osinowo, Matie Obazee and Henry Lawson. Africa has been a very difficult terrain for growing WONCA and is recognised as such by WONCA. WONCA had a memorable world onference in South Africa in 2001 and elected Bruce Sparks as its world president for 2004–2007. The first WONCA Africa conference was held in Nigeria in 2000, with a second conference held after many years in South Africa in 2009. This continued with Zimbabwe (2012), Ghana (2015) and South Africa (2017). Much of the recent success in holding conferences has come from Belgian support for African family medicine, especially the Primafamed Network and its collaboration with WONCA conferences.

**FIGURE 1:**
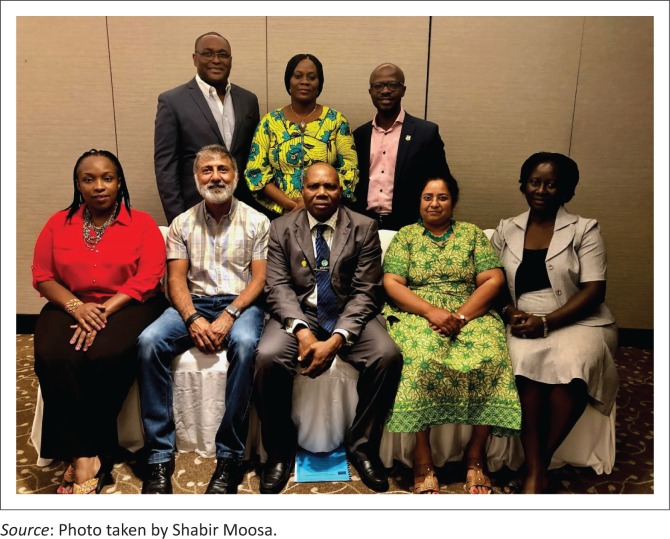
WONCA Africa Executive Committee – Seated from left to right: Joy Mugambi, Shabir Moosa, Dan Abubakar, Elizabeth Reji and Jane Namatovu. Standing from left to right: Henry Lawson, Temitope Ilori and Nana Kwame Ayisi-Boateng.

I was elected as president-elect at the WONCA Africa Council Meeting before the WONCA Conference in Rio de Janeiro, Brazil (2016) to serve under President Henry Lawson (2016–2018) and to then serve as President from 2018 to 2020. WONCA Africa Council met again before the WONCA Conference, Seoul, South Korea (October 2018) to elect the new WONCA Africa Executive Committee (see Figure 1): Dr Dan Abubakar from Nigeria as president-elect, Dr Joy Mugambi from Kenya as secretary, Dr Elizabeth Reji from South Africa as treasurer, Dr Temitope Ilori from Nigeria and Dr Jane Namatovu from Uganda as members-at-large and Dr Henry Lawson from Ghana as ex-officio past president. Dr Nana Kwame Ayisi-Boateng joins the Executive Committee as African chair. This represents one of the best teams in WONCA Africa, with strong women leadership.

There are many plans but the first priority is to ensure that the next WONCA Africa Conference in Kampala, Uganda, 06–08 June 2019, is a success. The website is available at www.WoncaAfrica2019.com. Register and come to Uganda to interact with an array of international speakers on the theme ‘People-centred PHC’. The year 2019 will be a momentous year, with the implementation of the Astana Declaration becoming a key issue amongst governments. We intend to engage with the WHO globally and in Africa to facilitate relationships between ministries of health and WONCA member organisations in Africa. We will be inviting the WHO Regional Director to speak at our Regional Conference and to meet leaders of our member organisations. There is a number of other plans: building membership of WONCA Africa, especially academic departments of family medicine; building resources for academic departments including research collaborations; improving women and young doctor participation in WONCA Africa and member organisations; strengthening African involvement in WONCA working parties and special interest groups; improving funding of WONCA Africa; adopting regional bylaws to ensure good governance of WONCA Africa; and improving communications with ordinary family doctors as well as member organisations.

This is the first editorial from WONCA Africa. There will be more details on these plans as we proceed. Check the WONCA Africa website at https://www.woncaafrica.org, and @WoncaAfrica on Facebook, Twitter and Telegram to keep abreast of things at WONCA Africa.

